# Tumor-Draining Lymph Node-Targeted Electrochemotherapy: A Hypothesis for In Situ Cancer Vaccination

**DOI:** 10.3390/biomedicines13112746

**Published:** 2025-11-10

**Authors:** Reihane Mahdavi, Hossein Ataee, Amirparsa Abdollahian Dehkordi, Mahdi Shabani, Azam Hemati, Mohammad Abdolahad

**Affiliations:** 1 Nano Bioelectronics Devices Lab, Cancer Electronics Research Group, School of Electrical and Computer Engineering, Faculty of Engineering, University of Tehran, Tehran 1439957131, Iran; 2 Cancer Electronics Research Center, Tehran University of Medical Sciences, Tehran 1416634793, Iran; 3 School of Medicine, Tehran University of Medical Sciences, Tehran 1419733141, Iran; 4 Department of Immunology, School of Medicine, Shahid Beheshti University of Medical Sciences, Tehran 1985717434, Iran; 5 Biotechnology Research Center, Pasteur Institute, Tehran 1316943551, Iran

**Keywords:** lymph node, immune suppression, metastasis, cancer immunotherapy, immunogenic cell death, electroporation, electrochemotherapy, abscopal effect

## Abstract

Immune checkpoint inhibitors (ICIs) have revolutionized cancer immunotherapy by enhancing T-cell-mediated anti-tumor responses in solid malignancies. However, their efficacy is often limited by tumor-specific factors, T-cell dysfunction in cold tumors, or in the presence of lymph node metastasis (LNM). Moreover, clinical trials indicate no significant survival advantage of sentinel lymph node biopsy (SLNB) over no lymph node surgery in early-stage cancers, highlighting the need for novel combinatorial approaches to improve treatment outcomes. Tumor electrochemotherapy (ECT) can overcome immunosuppressive barriers in the tumor microenvironment by applying high electric fields that create transient micropores in cell membranes. This allows the enhanced uptake of chemotherapeutic drugs, resulting in increased cytotoxicity and the release of damage-associated molecular patterns (DAMPs). This triggers immunogenic cell death (ICD), a process that signals immune cells to attack cancer and promotes tumor regression. Considering advancements in lymphatic-targeted therapies and the immunostimulatory potential of uninvolved tumor-draining lymph nodes (TDLNs), TDLN-targeted ECT may act as an in situ cancer vaccination, activating immune cells within TDLNs through the release of DAMPs and serving as a hub to orchestrate systemic anti-tumor immunity. In patients with negative preoperative lymph node assessments, this approach may preserve lymph node integrity and lymphatic drainage while eradicating tumor cell colonies. When applied as neoadjuvant therapy before any TDLN treatment, TDLN-targeted ECT may leverage higher tumor-associated antigen loads, foster persistent immune memory, and reduce the risk of post-operative immune evasion.

## 1. Introduction

Two primary circulatory systems exist in mammals: the cardiovascular and the lymphatic vascular systems (LVS). Lymph nodes (LNs), classified as secondary lymphoid organs (SLOs), play a vital role in the function of the immune and circulatory systems, as they connect both systems [1]. The fundamental function of the lymphatic system is to regulate fluid balance by collecting excessive interstitial fluid and proteins, such as antigens. Lymph also includes leukocytes that enter the lymphatic vessels via chemoattractant molecules and flow unidirectionally to LNs [1]. Later, the lymphatic system filters substances that pass through afferent lymphatic vessels to LNs and orchestrates the adaptive immune responses by activating naïve lymphoid cells through establishing effective interactions among lymphocytes and other functional immune cells, such as antigen-presenting cells (APCs) in the cortex and paracortex regions of the LNs. Consequently, transport activated lymphoid cells into the circulation [2].

In the case of solid tumor progression, Tumor-Draining Lymph nodes (TDLNs) are supposed to have two contradictory assignments:

(1) An “Arsenal” for holding a supply of immune agents to be fed automatically to the cancer battlefield. (2) A pathway for cancer development and progression due to tumor cells colonizing in TDLNs that raises concerns about the potential of distant tissue metastasis [3,4].

The mechanisms by which tumor cells enter TDLNs or navigate the lymphatic system rather than the bloodstream are not yet fully understood. Some studies suggest that fluid dynamics and interstitial fluid pressure (IFP) gradient in the tumor microenvironment (TME) may play a key role in tumor cells migration to the lymphatic system [5,6], It is also proposed in the literature that tumor cells are exposed to the lower levels of shear stress in the lymphatic system, as compared to the higher flow velocities evident in blood vessels, leading to the survival of these malignant cells in the lymphatic drainage and the subsequent TDLN invasion [7]. Regulation of lymph angiogenesis through the activation of Vascular Endothelial Growth Factor Receptors C and D (VEGFR-C/D) on lymphatic endothelial cells (LECs), triggered by VEGF-C/D released from cancer cells, is also a commonly proposed mechanism for cancer cell invasion into TDLNs [8]. It is while some other research proposes that chemoattractant molecules released by LECs may guide tumor cells toward lymphatic vessels [9].

A traditional theory named the Halstedian model proposed that tumors spread sequentially from lymphatics to locoregional or distant LNs and then to distant organs for metastasis [10]. Based on this theory, removing TDLNs reduces the likelihood of recurrence, which is expected to enhance both overall survival (OS) and progression-free survival (PFS) rates [11]. However, recent research challenges this view through animal and human studies, suggesting distant metastases may differ from those within TDLNs both phenotypically and clonally [12,13]. Moreover, the systemic model suggests that cancers spread to distant tissues through the bloodstream and that TDLN involvement does not contribute to tumor development and metastasis [14,15,16,17,18,19]. Although these assumptions have opened a new avenue of research into the relationship between cancer development and the lymphatic system, TDLN dissection still holds a special place in the main standards of cancer surgical management. Regarding the value of nodal status assessment in determining tumor prognosis [20], many guidelines recommend Sentinel LN biopsy (SLNB) even in the absence of confirmed involvement with malignant cells [21,22,23].

For example, in breast cancer surgery, SLNB is always performed. In cases where the sentinel node is involved by cancer cells, Axillary Lymph Node Dissection (ALND) is performed, and sometimes several negative, uninvolved LNs are also dissected (radical lymphadenectomy) [23]. However, ALND is associated with several complications, such as seroma formation, lymphedema, pain, limitations in arm movement, and loss of sensitivity [24]. While lymphadenectomy is commonly performed for staging and therapeutic purposes in various cancers, recent trials in advanced ovarian cancer [25], melanoma [26,27], breast [28], thyroid [29], gastric [30], and renal cell carcinomas [31] show little to no improvement in Overall Survival (OS) of patients in comparison to limited or regional lymphadenectomy. For instance, a recent study named the ACOSOG Z0011 randomized clinical trial (RCT) demonstrated that in T1 or T2 invasive breast cancer patients with one or two involved sentinel LNs in cancer, ALND, along with SLNB, does not provide a meaningful difference in the 10-year OS rate compared to SLNB alone (446 patients in the SLNB alone group and 445 in the SLNB + ALND group) [28]. Another similar RCT (SOUND) demonstrated that SLNB does not make a significant difference in OS or distant metastasis percentage with no axillary surgery in early-stage breast cancer patients having a tumor up to 2 cm in diameter and a negative result on preoperative US of the axillary LNs, indicating that patients in these settings could be safely spared from axillary surgery. (708 patients in the SLNB group and 697 in the no axillary surgery group, 98.2% vs. 98.4% 5-year OS, confirming that the omission of axillary surgery was non-inferior to SLNB) [32]. So, the therapeutic benefit of lymphadenectomy remains controversial [24,33].

In Addition, recent research highlights the importance of TDLNs in initiating effective responses and higher response rates to cancer treatments. For instance, immune checkpoint blockade immunotherapy activates T-cells in TDLNs, and it has been demonstrated that the absence of TDLNs leads to a significant decrease in treatment efficacy [33,34]. Another study found TDLN presence to be a critical mediator of the abscopal effect induced by radiotherapy, claiming TDLN serves as a stem-like CD8+ T-cell supplier and plays a key role in populating the tumor with CD8+ T-cells [35]. Nevertheless, current surgical guidelines instruct surgeons to remove TDLNs as part of the metastatic route, irrespective of their impact on postoperative treatment.

## 2. Immune Suppression Mechanisms in TDLNs

**Figure 1:**
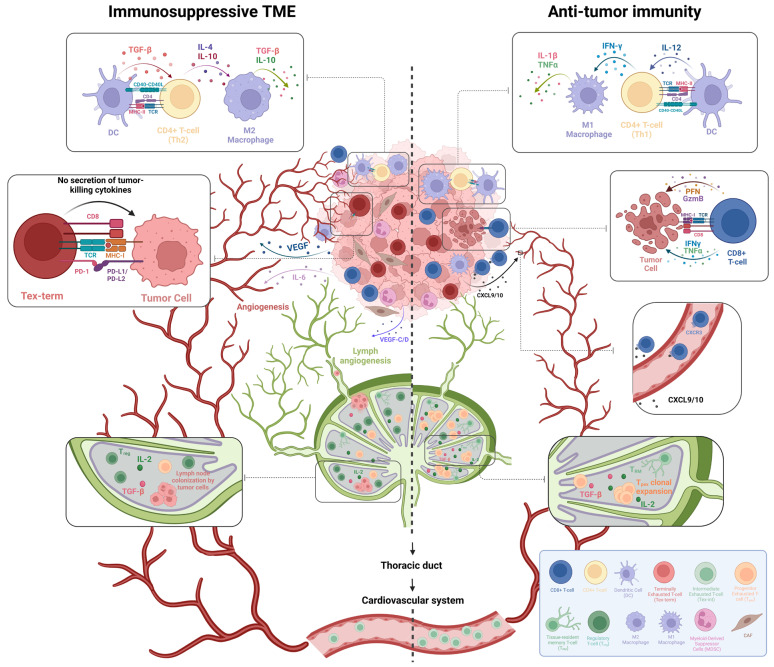
A comparison between an immunosuppressive TME versus anti-tumor immunity. Some properties are evident in anti-tumor immunity responses: Decreased inhibitory receptors expression on T-cells and terminally exhausted T-cells (T_ex-term_) count in TME, effective infiltration of cytotoxic T-cells and M1 macrophages in TME, which is associated with cytotoxicity and anti-tumor immunity, lack of myeloid-derived suppressor cells (MDSCs) infiltration in TME, decreased angiogenesis, CD4+ helper T-cells differentiating into Th1 cells, increased levels of IL-12, IFN-γ, IL-1b, TNF-α and, granzyme B (GzmB), increased progenitor exhausted T-cells (T_pex_) proliferation in TDLNs, increased IL-2 level in TDLNs, increased tissue-resident memory T-cells (TRM) in TDLNs, decreased regulatory T-cells (T_regs_) count in TDLNs, and increased intermediate exhausted T-cells (T_ex-int_) count in blood circulation. On the other hand, different properties are noticeable in an immunosuppressive TME: increased inhibitory receptors expression, which are abundant on T_ex-term_, increased angiogenesis, increased MDSCs infiltration in TME, CD4+ helper T-cells differentiating to Th2, high levels of TGF-β, IL-4, IL-6 and IL-10 in TME, tumor-promoting M2 macrophages in TME associated with tissue healing and neoantigen tolerance, increase in T_regs_ count in TDLNs, decrease in T_pex_ proliferation and TRM count in TDLNs, decrease in IL-2 level in TDLNs, and decrease in T_ex-int_ cells count in blood circulation (“Created in BioRender. A.A.D. (2025) https://BioRender.com/5gx818o, accessed on 24 October 2025”).

T-cells, crucial mediators of adoptive immunity, might undergo different fates after antigen recognition. One possible fate is exhaustion, a form of suppression that occurs in CD3+ lymphoid cells during chronic infection or cancer [36]. Exhausted T-cells exhibit characteristics such as a decrease in cytokine secretion, an increase in inhibitory molecule expression, and reduced proliferative capacity [37]. To add more context, T-cells can be at different stages of exhaustion, ranging from progenitor-exhausted T-cells (T_pex_) to intermediate-exhausted T-cells (T_ex-int_) and terminally exhausted T-cells (T_ex-term_), each with its own characteristic tendencies [38]. Suppose that T_pex_ and T_ex-term_ are usually tissue-resident subsets, while T_ex-int_ cells are mostly circulating, with T_pex_ and T_ex-term_ cells being abundant in TDLNs and TME, respectively [39]. Exhaustion arises when T-cells are excessively exposed to antigens, which can happen in any region where active antigen-presenting cells (APCs) are in close proximity to T-cells. This setting includes the tumor microenvironment (TME), where mainly dendritic cells (DCs) present antigens to T-cells [40]. The binding between the antigen on the major histocompatibility complex (MHC) of DCs and the T-cell receptors (TCRs) might exhibit high, moderate, or low affinity. High-affinity interactions, alongside CXCL4 downregulation, result in T-cells being retained in the TME [41], which ultimately leads to their exhaustion. Nevertheless, a low or moderate affinity interaction shields T-cells from nearby dysfunction, prompting their egress from the TME towards the TDLN(s) via CXCL 12 decrease [41] and Sphingosine 1-Phosphate Receptor 1 (S1PR1) on T-cells [42]. The retention of T-cells in TDLNs relies on transforming growth factor β (TGF-β) signaling [43]. This signaling and the presence of tissue-resident memory T (TRM) cells form a significant reservoir of antigen-specific immunity [1] (Figure 1).

Like T-cells, tumor cells can pass through the lymphatic system or colonize TDLNs [44] (Figure 1). This colonization is accompanied by lymph angiogenesis and the development of tumor-specific immunological tolerance, which in turn makes distant organs susceptible to metastasis. The induction of immunological tolerance occurs through driving the differentiation of naïve CD4+ T-cells into regulatory T-cells (T_regs_) in the immunosuppressed TDLNs, which then dampen the immune response in an antigen-specific manner [45] (Figure 1). Furthermore, there exist numerous other mechanisms that induce immunosuppression, such as increased population of dysfunctional T-cell subsets which lack effector functions [46,47], metabolic alterations in the TME that can lead to suppression of T-cell responses by depriving essential nutrients [48], the presence of tumor-derived VEGF-C linked to metastatic advancement [49], and heightened expressions of PD-L1 (Figure 1) [50].

## 3. Immunotherapy: Reinvigorating the Immune System

Since 1960, many efforts have been made to enhance the immune system’s ability to identify and battle cancer progression, a field known as immunotherapy [51]. In this regard, James P. Allison and Tasuku Honjo were awarded the Nobel Prize in medicine in 2018 for the development of a groundbreaking cancer immunotherapy approach called immune checkpoint inhibitors (ICIs) [52]. Checkpoints are molecules expressed on different leukocytes. Upon binding to their ligands, they exert immunosuppressive effects through diverse mechanisms. While many checkpoints are expressed on different immune cells, the most notable ones are PD-1 and its ligand PD-L1, CTLA-4 and its ligand CD80/86, and TIM-3 and its various ligands, such as Gal-9 and HMGB1 [53]. Above, we pointed out an immunosuppressive mechanism by which tumors increase PD-L1 expression in tumor cells. PD-L1, an immune checkpoint ligand expressed on macrophages, activated T-cells, B-cells, DCs, cancer cells, and some epithelial cells under inflammatory conditions, suppresses the immune system by binding the PD-1 molecule on T-cells. This interaction between two molecules functions as a “brake” on T-cells, inhibiting effector function and, ultimately, leading to cell apoptosis [54].

ICIs are substances that attach to the checkpoints, preventing them from binding to their respective ligand(s). This action eventually enhances the immune response [55]. Different checkpoints are present in different immune system interactions. For example, PD-1 is involved in the interaction between T-cells and tumor cells, while CTLA-4 inhibits T-cell activation during the interaction with DCs by binding CD80 [56]. Recently, a considerable number of ICIs for different checkpoints have been FDA-approved for many cancers, including melanoma, non-small cell lung cancer (NSCLC), head and neck squamous cell carcinoma (HNSCC), triple-negative breast cancer (TNBC), Renal Cell Carcinoma (RCC), etc. These drugs include Pembrolizumab and Nivolumab targeting PD-1, Ipilimumab targeting CTLA-4, and Atezolizumab targeting PD-L1 [57,58]. Additionally, Pembrolizumab has been approved for Microsatellite Instability-High (MSI-H) solid tumors [59] and Tumor Mutational Burden-High (TMB-H) solid tumors [60], making it the first cancer drug to date approved regardless of cancer type and based on a molecular biomarker [61]. Numerous studies conducted in recent years have substantiated the importance of ICIs. These studies have demonstrated that when ICIs are used in combination with other cancer treatments, such as chemotherapy and radiotherapy, or alone as monotherapy, they improve anti-tumor responses in different cancers [62,63,64,65,66,67].

## 4. Cold vs. Hot Tumors: Why Many Fail to Respond to ICIs

Despite all the benefits of immunotherapy, it is not consistently effective as a cancer treatment modality, and numerous potential pitfalls can lead to its failure [68]. There are two tumor subsets based on response to ICIs: cold and hot tumors. In hot tumors, the effective presentation of antigens, T-cell clonal expansion, T-cell activation, and, consequently, induction of PD-L1 production on tumor cells by IFN-γ are found, which leads to a favorable ICI immunotherapy response [69]. In these cases, other factors that boost anti-tumor immunity in TME, such as the M1 macrophages [70], Th1 cells [71], an increase in anti-tumor cytokines like IFN-γ and granzyme B (GzmB) [72,73,74], increased CXCL9/10 chemokines which correlate with lymphocyte infiltration [75], contribute to an overall better immune response (Figure 1).

However, immunotherapy is ineffective in cold tumors due to either T-cell desertification or T-cell exclusion, resulting in a poor response [76]. T-cell desertification refers to a condition in which T-cells are absent from the TME and surrounding tissues due to physical barriers or immunosuppressive molecules. As a result, T-cell desertification resembles a “tumor ignorance” state in which the immune system, and T-cells fail to interact with tumor cells [77]. Consequently, in the absence of PD-1/PD-L1 interaction, ICI response is almost absent in these tumors [78]. In T-cell exclusion, presenting antigens and the subsequent clonal expansion of T-cells occur normally. However, lymphocytes and exclusively activated T-cells cannot infiltrate the tumor core properly [79] due to some TME elements such as myeloid-derived suppressing cells (MDSCs) [80], Extracellular matrix [81], Tumor-associated stroma [82], cancer-associated fibroblasts (CAFs) [83], and increased inhibitory cytokines such as TGF-β, IL-6, and IL-10 [84] (Figure 1). Therefore, T-cells cannot interact with the core of the tumor and exert their anti-tumor functions, leading to the failure of ICI [76]. There is an ongoing debate on which biomarkers should be measured to predict a tumor’s response to immunotherapy, but researchers have demonstrated that markers such as the presence of mature Tertiary Lymphoid Structures [85] (mTLS), high PD-L1 expression [86], high TMB [87], and high MSI [88] are positively associated with immunotherapy responses. Meanwhile, factors that can impair immunotherapy outcomes include a β2 microglobulin mutation in tumor cells [89], increased expression of other inhibitory checkpoints on terminally exhausted T-cells [90], and tumor cells’ resistance to IFN-γ [91]. This resistance prevents PD-L1 induction on tumor cells and results in T-cell suppression in a PD-1/PD-L1 independent manner, which cannot be rescued by blocking the PD-1/PD-L1 axis alone [92]. Besides these potential causes for immunotherapy failure, a recently discovered culprit is the absence of an effective TDLN immune response. Recent research on TDLNs in the context of immunotherapy has uncovered new insights into their role and potential to enhance the efficacy of treatments [34,93].

## 5. The Pivotal Role of Uninvolved TDLNs in Initiation of ICI Response

A recent study on CD8+ T-cell responses to neoadjuvant anti-PD-L1 immunotherapy analyzed distinct exhausted T-cell subsets (T_ex_) abundance in human regional uninvolved TDLNs (uiLNs) compared to metastatic ones (metLNs) [94]. This study claims that T_pex_ cells in uiLNs are clonally related to T_ex_ cells in TME. This result demonstrates that LNs are a crucial reservoir for functional CD8+ T-cells, which are an important target in immunotherapy.

According to the results, after ICI, the frequency of both T_pex_ and T_ex-int_ changed in uiLNs, with T_pex_ having the most significant decrease among CD8+ subsets, while T_ex-int_ had the most significant increase among CD8+ subsets. Regarding proliferation, the percentage of Ki-67+ cells in the T_pex_ subset did not change, whereas the percentage of Ki-67+ cells among the T_ex-int_ subset increased significantly. Not only in uiLN but also in blood, the T_ex-int_ subset showed a significant increase in both frequency and Ki-67 expression. However, in TME, little proliferation of the T_ex-int_ subset was evident. Also, the increased frequency of T_ex-int_ in the tumor was positively associated with a decrease of T_pex_ and an increase of T_ex-int_ in uiLN, consistent with the origin of the T_ex-int_ subset, which is derived irreversibly from T_pex_. Based on the evidence, we hypothesize that a successful treatment modality is beneficial for tumor control when it promotes high proliferation of circulating T_ex-int_ cells and the accumulation (or diffusion) of effector T-cells in the TME, while maintaining an adequate T_pex_ population in the TDLNs.

On the other hand, the effect of ICI on CD8+ cell composition within metLNs differs markedly from that in uiLNs and is almost the same as the TME, with T_pex_ cells being nearly absent, and T_ex-term_ cells abundant. Furthermore, T_ex-int_ and T_pex_ are more localized around immunosuppressive niches in metLNs. T_regs_ with higher proliferation capacity, overexpression of regulatory molecules, including master regulator transcription factor (FoxP3) [95], and DCs with higher levels of regulatory molecules (IDO, CD39, TIM-3, and PD-L1, which are all associated with tolerogenic DC state) [96,97] are the characteristics of such an immunosuppressive TME. Compared to cancers confined to the primary site, patients with metLNs showed a smaller increase in both frequency of T_ex-int_ and Ki-67+ T_ex-int_ t in the blood. These data suggest that the alterations in T_pex_ frequencies and cellular neighborhoods in metLNs are linked to reduced reactions to ICIs in the bloodstream [94]. In conclusion, according to this article, uiLNs play a pivotal role in the initiation of the anti-tumor immune response, and this crucial effect can be impaired with the colonization of tumor cells in TDLNs.

## 6. Emerging LN-Targeted Immunotherapies (Drugs, Vaccines, Nanoparticles)

Targeting lymphatic vessels and LNs for immunotherapy has been in the spotlight recently [98,99,100,101]. Delivering ICI drugs directly to the LNs is a developing modality for immunotherapy [102]. The effectiveness of systemic administration of ICIs in treating tumors is hindered by the low concentration of therapeutic antibodies in the specific tissues where the immune response against tumors occurs, such as the TME and secondary lymphoid organs. Compared to systemic administration, intradermal or intratumoral delivery of ICIs results in a greater concentration in both the TME and its associated LNs, leading to a strong immune response, immunological modulation in the LNs, effective tumor growth suppression, and minimizing the toxicity issues commonly associated with systemic and high-dose ICI therapies [102]. For example, evidence indicates that directing treatment towards the TDLNs in mice models of melanoma and breast cancer leads to the development of strong immune responses against tumors, surpassing the effectiveness of systemic administration [102].

In addition to ICIs, scientists are exploring other options, such as LN-targeted vaccines [103]. These include direct delivery of tumor-specific antigens to LNs to enhance the effectiveness of tumor vaccines using carrier molecules, such as functionalized liposomes or nanoparticles [104]. These carrier molecules can be modified by targeting ligands or antibodies to increase their specificity for LNs. Innovative approaches for delivering desired agents to LNs, such as microneedles, have recently become a popular research subject [105]. These nano-vaccines can reach LNs by gradual diffusion and tissue movement, or they can be carried by APCs like DCs or macrophages [106]. So, they can trigger a strong and long-lasting immune response against metastatic cancer cells [107]. These findings emphasize the importance of focusing on LNs, especially TDLNs, as a crucial site of the immune system for developing novel cancer therapies.

## 7. Clinical Controversies in Lymph Node Surgery: To Remove or Preserve TDLNs?

**Figure 2:**
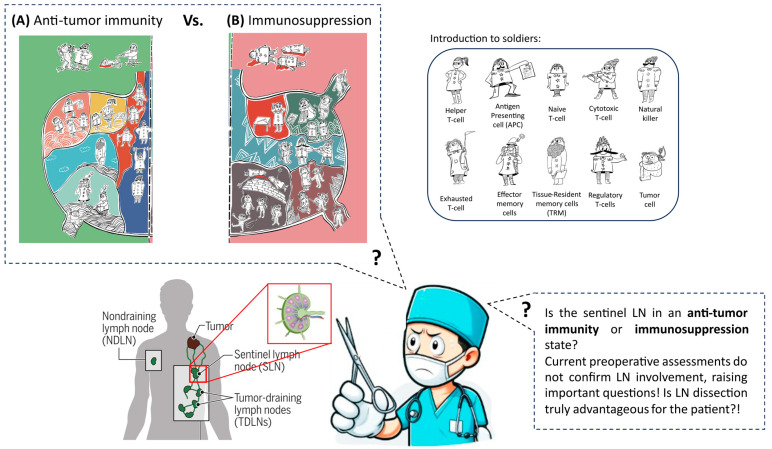
Cartoon depiction of two possible states of TDLNs according to the disease progression in patients with negative preoperative results, making the surgeon confused. These two possible states may include immunosuppression or anti-tumor immunity. TDLNs’ environment is analogous to an arsenal invaded by foreign enemies, and soldiers are defeating the arsenal. There are many differences between immunosuppressed and active TDLNs. In activated TDLNs with anti-tumor functions (**A**), we have effective antigen presentation by APCs, non-exhausted and proliferative cytotoxic T-cells, active helper T-cells, and also other important immune cells such as tissue-resident memory T-cells (TRM), NK cells, and effector memory T-cells. This cell composition in TDLNs leads to effective anti-tumor immunity in the bloodstream and TME, as demonstrated by NK and CD8+ T-cells destroying tumor cells outside the TDLN environment. On the other hand, in immunosuppression (**B**), many differences are spotted, such as tumor cell colonization of TDLNs, lymph angiogenesis, lack of antigen presentation by APCs, exhaustion in CD4+ and CD8+ T-cells, and an abundance of T_regs_ in TDLNs, which leads to effector T-cell apoptosis. Due to TDLNs’ pivotal role in systemic immunity, all these factors combine to yield a weak anti-tumor immune response.

Findings described in previous sections have recently raised some ambiguities in the surgical management of cancer, related to the complicated nature of tumor and TDLNs interaction in different stages of the disease progression. TDLNs removal may be a serious source of confusion for a surgeon, especially in patients with a negative preoperative LN involvement that usually does not provide precise data about the LN involvement in cancer (Figure 2). According to guidelines, the surgeon should dissect TDLNs to obtain a more accurate LN diagnosis in the setting of intraoperative frozen-section, leading to permanent loss of the sentinel LN and a possible therapeutic opportunity for the patient. The surgeon is aware of the undeniable role of TDLNs in the initiation and maintenance of anti-tumor immune-related responses in the body and their constitutive effect in postoperative treatments such as immunotherapy and radiotherapy, as mentioned before [35,94]. Nevertheless, the surgeon may be worried about the role of TDLNs as a possible metastatic route. Despite the potential of TDLNs, this concern alone often leads to their removal, subjecting the patient to the side effects of radical LN surgery, such as long-lasting lifestyle changes due to physical restrictions, fatigue, and lymphedema [108]. However, a potentially significant therapeutic opportunity might also be lost, as these TDLNs—if preserved—could play a possible role in enhancing the body’s immune response against cancer. By removing them without fully exploiting their potential, the patient with no confirmation of LN involvement may miss an important avenue for improving treatment outcomes (Figure 2).

Despite these numerous disputes, a single question remains controversial. Is there any solution to preserve and boost these immune system “arsenals” and use their potential to fight cancer progression while maintaining the integrity and steady state of the lymphatic organs?

## 8. TDLN-Targeted Electrochemotherapy as an In Situ Vaccine: A Hypothesis

Despite the critical role of TDLNs as the key player in adaptive immune responses, their anti-tumor immune efficacy is gradually diminished by the incidence of cancer cell invasion and colonization in TDLNs, raising concerns about the conversion of TDLNs to a metastatic route for cancer progression, which ultimately leads to inevitable LN surgeries. However, recent studies have begun questioning the benefits of radical LN dissection for OS, PFS, and cancer recurrence. These insights underscore the importance of discovering a way to restore TDLNs to their efficient functions, one of which may be TDLN-targeted treatments and stimulations to eradicate cancer cells, trigger immune responses, and possibly even enhance the effectiveness of standard treatments such as immunotherapy.

Electrochemotherapy (ECT) is an innovative solid tumor therapy that uses electroporation in combination with chemotherapy drugs. Electroporation enhances cell membrane permeability by applying a set of milli- to nano-second pulsed electric fields [109]. When the cell membrane is exposed to a high electric field, water pores form and water molecules penetrate the membrane phospholipid bilayer. As a result, the adjacent lipids rearrange, aligning their polar head groups towards the water molecules. The process is amplified by the creation of a Transmembrane Potential (TMP), which results from membrane polarization [110]. Electroporation can be reversible or irreversible based on the characteristics of the electric pulse and the targeted cell’s physiological properties. In irreversible electroporation (IRE), cells are exposed to a strong electric field, disrupting their homeostasis and leading to cell apoptosis. Consequently, IRE eradicates a considerable volume of tumor tissue while avoiding harmful thermal effects. In reversible electroporation (RE), moderate electrical pulses temporarily increase cell membrane permeability, allowing molecules to enter cells while maintaining cell survival. This method facilitates the delivery of functional genetic components or drugs to targeted cells, having a broad application in genetics and drug delivery techniques [110].

ECT combines RE with chemotherapeutic agents, commonly bleomycin and cisplatin. These drugs are highly toxic and can cause different types of cell death, with the outcome depending on the drug concentration within the cell. Low drug concentrations cause cell-cycle arrest, while high concentrations lead to DNA fragmentation and pseudo-apoptosis. RE enhances the uptake of these drugs by tumor cells, increasing their cytotoxic effects [111]. For example, according to the literature, bleomycin and cisplatin uptake are increased by about 100- to 700-fold in ECT. Moreover, ECT allows for prescribing lower doses of these drugs, minimizing side effects and improving patient tolerance [112,113]. ECT has been subjected to clinical trials since the early 1990s, targeting various cancers such as melanoma, sarcoma, breast cancer, renal cell carcinoma, liver cancer, and pancreatic cancer. After more than thirty years of extensive research and clinical evaluations, it has been included in the official treatment guidelines for melanoma, Basal Cell Carcinoma (BCC), and Squamous Cell Carcinoma (SCC) [114,115,116,117,118].

In recent years, a significant additional aspect of electrochemotherapy has emerged: its influence on the immune system. Therapies that utilize ECT and IRE induce a controlled form of cell death termed immunogenic cell death (ICD). This process is marked by the release of specific molecules called Damage-Associated Molecular Patterns (DAMPs), which include adenosine triphosphate (ATP), calreticulin (CRT), and the high-mobility group box 1 (HMGB1) protein. These molecules signal the immune system to attack the cancer cells by facilitating the capture and presentation of tumor antigens by APCs [119]. Cell death-inducing electroporation techniques, including ECT and IRE, are not limited to treating local tumor sites; they can also provoke a systemic immune response that may target distant tumors. They possess the ability to quickly release DAMPs and tumor-associated antigens (TAAs) while maintaining the integrity of tumor blood vessels, which is crucial for immune cells to access and attack the tumor. It also reshapes TME to support a better immune response [120]. Thus, these therapies act as in situ vaccines by inducing immunogenic cell death, thereby enhancing the systemic antitumor response [110]. This effect could be further exploited by combining immunotherapy. Clinical trials of ECT combined with immunotherapy demonstrate promising results in hepatocellular carcinoma (HCC), melanoma, and breast cancer [121,122]. However, further comprehensive trials are still required.

In addition, several research groups have explored the treatment of inoperable recurrent LNs through the application of ECT and IRE. These studies approached LNs solely as sites of metastasis, not as immune organs, aiming to eliminate cancer cells within them. They reported successful clearance of these inoperable LNs, including those that were bleeding or significantly enlarged, and noted a considerable reduction in their size [123,124]. Another study applied IRE to LNs in an animal model, demonstrating that the LN structure remained intact even when subjected to a high electric field, and no detectable damage to the LN capsule was observed in radiological and histopathological evaluations [125]. Also, in vitro ECT on immune cells shows the potential to preserve immune cell integrity during electrochemotherapy [126].

**Figure 3:**
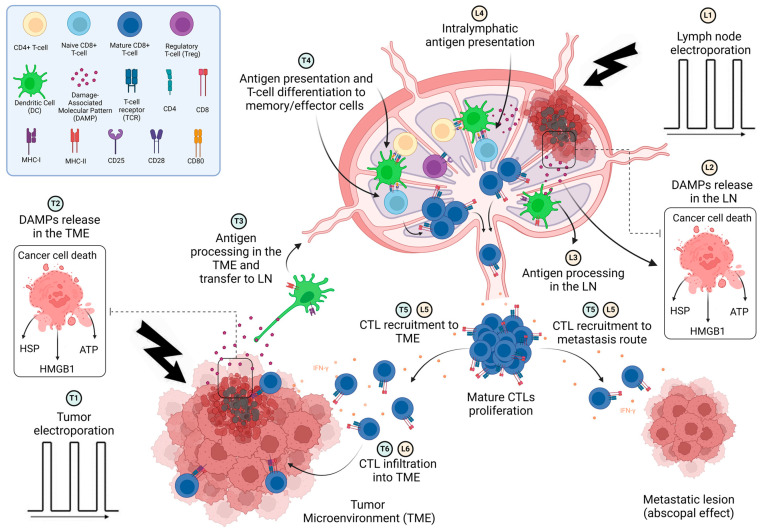
Chain of possible immune-related events happening in concurrent tumor- TDLN DAMPs induction by ECT. ‘L’ is the term used for immune-related events initiated from TDLNs ECT, and ‘T’ refers to tumor-related ECT events. TDLNs ECT (L1) results in DAMPs’ release in the TDLN environment (L2), in close contact with immature immune cells. Both antigen encounter (L3) and presentation to naïve T-cells via APCs are performed within the TDLN in the presence of CD4+ helper T-cells, leading to production of mature Cytotoxic T lymphocytes (CTLs) (L4). CTL recruitment to the TME and metastasis route (abscopal effect) is then performed (L5, L6). On the other hand, tumor cell ECT (T1) leads to DAMPs production in the TME (T2), which promotes APCs migration to TME and antigen processing (T3). T-cell differentiation to CTLs is subsequently performed in the TDLNs environment in the presence of helper T-cells, leading to clonal expansion (T4), CTL recruitment to TME (T5), and infiltration into the tumor core (T6) (Created in BioRender. A.H. (2025) https://BioRender.com/lvx9dit, accessed on 24 October 2025”).

Given these findings, a critical inquiry emerges: could the TDLN-targeted electrochemotherapy help with the better management of patients with no preoperative confirmation of LN involvement? Does it bolster the anti-tumor immune response, and if so, how? A possible scenario may include that applying ECT in colonies of cancer cells in the TDLNs, induce ICD in close contact with a reservoir of immune compartments and may accelerate the orchestration of anti-tumor immunity cycle (i.e., antigen encounter with APCs, effective antigen presentation, clonal expansion of related immune cell subsets such as T-cells, and the whole immune response) (Figure 3). This could potentially strengthen the anti-tumor immune response and improve the quality of the immune reaction. It also clears targeted TDLNs of cancerous cells, lowering immune tolerance and overcoming immunosuppressive obstacles, allowing more effective immune-related therapies such as ICI.

Additionally, concurrent ECT of both the tumor and the TDLNs could result in more effective antigen release and presentation (Figure 3). Considering that ECT techniques can induce tissue shrinkage, it is likely that this approach may also diminish some immunosuppressive obstacles and change cold tumors to hot ones. Indeed, DAMP induction by ECT may compensate for the lack of TMB in the case of T-cell desertification, in addition to increasing tumor-infiltrating lymphocytes (TIL) in the case of T-cell exclusion in the TME. Figure 3 depicts the possible immune-related events associated with the concurrent application of ECT in TDLN and TME. As illustrated in the picture, T-cell activation and clonal expansion are performed via two different routes. First, antigen recognition in the TME and antigen presentation within the TDLNs. In the second scheme, both antigen processing and its presentation to naïve T-cells are performed in the TDLN environment.

## 9. Discussions

Achieving cancer cell-specific cell death colonized in TDLN while preserving alive and functional immune cells is essential for TDLN-targeted ECT. Immune cells may respond to ECT differently due to factors such as smaller size and distinct membrane composition. The selective vulnerability of tumor cells to electric fields can be elaborated via Schwan’s equation [127,128], which describes the induced transmembrane potential (ΔVₘ) as proportional to the product of cell radius (r) and the external electric field strength as follows:

ΔVₘ = 1.5 E r cos(θ)

This equation indicates that larger cells reach the critical TMP for electroporation at lower external field strengths than smaller cells. Consequently, tumor cells that are approximately twice the diameter of lymphocytes and have heterogeneous, permeable membrane compositions are more likely to undergo membrane porosity and subsequent cytotoxic effects under the same field conditions. This biophysical principle supports the possibility of applying optimized electric fields to induce reversible or selective electroporation in tumor cells within TDLNs, while minimizing damage to surrounding immune cells.

To optimize the procedure, several strategies can be employed, including fine-tuning electric field parameters of field strength, pulse duration, and frequency, to selectively permeabilize tumor cell membranes while sparing immune cells, and combining ECT with immunomodulatory agents, such as cytokines or checkpoint inhibitors, to enhance anti-tumor immunity and support immune cell activation within TDLNs.

In this manner, the chemotherapeutic drug uptake is increased in tumor cells, following the release of DAMPs. Enhancing tumor mutational burden (i.e., releasing intracellular bodies like mRNA, ATP, HMGB1, Calreticulin, and Heat shock proteins in the lymph node microenvironment) triggers the immune system in two different ways of activating antigen-presenting cells (APCs) and raising the tumor antigen concentration in the lymphatic vessels as a systemic circulating route. While T-cell exhaustion in the TME is mentioned in the article as a potential reason for immune suppression, we believe that TDLN-targeted ECT could take advantage of priming non-exhausted T-cells in the TDLN instead and regulate them through the lymphatic circulatory system.

In addition to electric-field optimization, careful selection of the chemotherapeutic agent, its dose, and timing in combination with electroporation is critical for achieving maximal efficacy and safety. The choice of drugs such as bleomycin, cisplatin, or immune modulators should possess cytotoxic potency and the ability to induce ICD within LNs. The route of drug administration (e.g., intravenous, intratumoral, intraperitoneal) determines the kinetics of drug accumulation in TDLNs, and it is essential to ensure sufficient drug concentration in the TDLN at the time of electroporation. Therefore, Systematic preclinical studies are needed to define the optimal combination of electric field parameters, drug selection, dose, and timing to safely and effectively implement TDLN-targeted ECT.

Accurate electrode placement around TDLNs is also essential, especially for deep or delicate nodes. Image-guided techniques such as ultrasound or CT scan enable real-time visualization and precise electrode positioning. Some studies have also reported the modification of surgical multielectrode instruments, such as biopsy [129] and endoscopic [130,131] forceps, to serve as specialized electrode probes for targeted electroporation procedures. Emerging navigation methods, including electromagnetic tracking [132,133] and contrast-enhanced lymphography [134], further improve targeting accuracy. These advances enhance the safety and efficacy of TDLN-targeted ECT while preserving immune structures.

In our opinion, concurrent application of ECT in both TDLN and TME could pave the way for groundbreaking advancements in cancer therapy, especially in patients with no evidence of LN involvement in preoperative assessments. However, further comprehensive research is still required.

## 10. Conclusions

In light of the aforementioned details, considering TDLNs as targets for innovative treatments (such as ECT) could open up a new area of research. This approach may augment the immune response to cancer in the short term and may be especially valuable for patients with negative preoperative results. It may even transform the current practice of Sentinel LN dissection in these patients. Further animal and human studies are required to consider and confirm this hypothesis.

## Data Availability

No new data were created or analyzed in this study.
